# Retrospective Analysis of Glioblastoma Outcomes

**DOI:** 10.7759/cureus.62462

**Published:** 2024-06-16

**Authors:** Razvan Onciul, Corneliu Toader, Luca-Andrei Glavan, Razvan-Adrian Covache-Busuioc, Bogdan-Gabriel Bratu, Horia-Petre Costin, Antonio-Daniel Corlatescu, Alexandru Vladimir Ciurea, Matei Grama, Andreea-Anamaria Idu

**Affiliations:** 1 Department of Neurosurgery, University Emergency Hospital of Bucharest, Bucharest, ROU; 2 Department of Neurosurgery, Carol Davila University of Medicine and Pharmacy, Bucharest, ROU; 3 Department of Vascular Neurosurgery, National Institute of Neurology and Neurovascular Diseases, Bucharest, ROU; 4 Department of Neurosurgery, Sanador Clinical Hospital, Bucharest, ROU; 5 Department of Software, Syndical.io, Bucharest, ROU

**Keywords:** recurrence, survival, prognostic factors, surgery, glioblastoma

## Abstract

This retrospective mono-center study focuses on 144 cases of glioblastoma treated over a time span of 12 years in our clinic in Romania. We offer critical insight into the dreadful aspect of this tumor by highlighting the principal characteristics such as localization, the genetic information of each case, progression-free survival (PFS), and overall survival (OS). A tenth of our patients underwent a second surgical procedure, providing a comparable OS to the other part of our study group, proving that surgical treatment as salvage therapy is a viable option. Also, our research reinforces the fact that utilizing the Karnofsky Performance Scale is a great predictor of patient outcomes in glioblastoma patients. Even though radiotherapy and chemotherapy have mild effects in the context of this oncological disease, our research shows that O^6^-methylguanine-DNA methyltransferase (MGMT) methylation status and epidermal growth factor receptor (EGFR) amplification have an important effect on OS. Moreover, the particularity of our study, that our patients did not start adjuvant therapy right after surgery, highlighted by a low OS compared to the international literature, sheds light on the fact that chemotherapy and radiotherapy must be started right after the surgical procedure, according to the Stupp protocol. To sum up, our research takes into consideration the factors that influence patient survival and outcome in the battle against glioblastoma.

## Introduction

Glioblastoma (GBM) is one of the most aggressive brain cancers, with an average overall survival rate of about 15 months after diagnosis, even with current treatments [1]. The latest 2021 WHO classification of brain tumors has categorized GBM, isocitrate dehydrogenase (IDH)-wildtype, as a grade 4 central nervous system (CNS) tumor [2]. With an incidence rate of 3.19 per 100,000 people in the United States and a median age of 64 years, GBM is rare in children. The incidence is 1.6 times higher in males than in females and 2.0 times higher in Caucasians compared to Africans and African Americans, with lower rates in Asians and American Indians. GBM is typically found in the supratentorial region (frontal, temporal, parietal, and occipital lobes) and is rarely located in the cerebellum [3].

So far, very few risk factors have been linked to the development of GBM. Exposure to ionizing radiation, nuclear radiation, vinyl chloride, pesticides, smoking, and working in petroleum refining or rubber synthesis are some factors that may contribute to the development of GBM [3,4].

The treatment of GBM is complex, highly personalized, and very expensive. The cumulative average cost of GBM therapy is currently estimated to be about 95,000 USD per patient [5]. Firstly, radiological tests must be done, such as CT scans and MRI, followed by pet-CT scans [6]. Consequently, molecular markers are routinely tested in GBM, markers such as epidermal growth factor receptor (EGFR), O6-methylguanine DNA methyltransferase (MGMT), and tumor suppressor protein TP53 [7].

The standard protocol for GBM involves attempting a complete surgical resection, which is challenging because GBM is a very infiltrative tumor. This often leads to incomplete resection due to the risk of neurological deficits from removing important brain structures [8]. As a result, a supratotal resection is the most optimal surgical treatment for GBM, although it cannot always be achieved [9,10]. After surgery, the standard protocol continues with treatment using temozolomide (TMZ) and radiotherapy (RT) [11,12].

Despite global efforts to improve patient survival, the prognosis for GBM remains very poor.

## Materials and methods

Our research is based on GBM patients treated in the National Institute of Neurology and Neurovascular Diseases in Bucharest, Romania, in the Department of Neurosurgery between 2012 and 2024. Each patient was evaluated on multiple variables, including age, gender, location of the tumor, IDH, Karnofsky Performance Status (KPS), overall survival (OS), and progression-free survival (PFS). Furthermore, tumor recurrence and second surgical procedures were taken into account. The conditions of admission for patients in this retrospective study were patients over 20 years of age, follow-up available for them, and patients should have had a surgical intervention.

The research aligns with the main principles in the Declaration of Helsinki and obtained approval from the Ethics Committee of the National Institute of Neurology and Neurovascular Diseases in Bucharest, Romania (approval number: 5193/16.05.2024). Clinical data including risk factors and surgical interventions, as well as follow-up details such as postoperative complications and mortality rate, were extracted from relevant files. The processing of all data was done in compliance with the current General Data Protection Regulation (GDPR), and informed consent was obtained from all patients included in this study.

Statistical analysis and figure plotting were conducted using Python version 3.10, developed by the Python Software Foundation, located in Beaverton, Oregon, United States. The analysis involved the use of Python libraries such as Pandas, NumPy, Seaborn, and Matplotlib.

Surgical protocol

The preoperative preparation involved a thorough evaluation and detailed planning to ensure optimal surgical outcomes. This phase began with the assessment of the KPS score to evaluate the patient's functional status. A comprehensive neurological examination was conducted to establish a baseline for postoperative comparison. Essential paraclinical tests were performed, including a chest X-ray, a complete set of blood tests, and an EKG. In certain cases, a thoracic-abdominal-pelvic CT scan was required.

Detailed brain imaging was obtained using MRI with and without contrast. If significant cerebral edema was present, cerebral depletive agents were administered. Antiepileptic drugs were prescribed as necessary to manage and prevent seizures.

A crucial aspect of preoperative preparation was the detailed discussion with the patient and their family, weighing the risks and benefits of the surgery. This included considering the quality of life versus survival duration. The decision regarding the aggressiveness of the tumor resection was made by the patient and their family based on the surgeon's presentation of the relevant surgical variables.

Regarding the surgical procedure, neuronavigation and a surgical microscope are mandatory tools to ensure precision and safety during surgery. Small craniotomies centered on the lesion were performed using neuronavigation, adjusted according to the lesion's location and associated edema. The tumor was excised entirely using the piecemeal technique under microscopic guidance. Intraoperative neuromonitoring and fluorescence were not utilized due to financial constraints. Without the use of fluorescence enhancing technique, gross total resection (GTR) was achieved in all surgically treated patients that were discussed in this study.

Specimens were sent for frozen section examination, with efforts made to collect samples from both the periphery and the central area of the lesion. Upon completion of the surgery, specimens were sent for paraffin-embedded histopathological examination. The bone flap was either reattached, or cranioplasty was performed using a titanium mesh, depending on the surgical approach and findings. Postoperatively, the patient was transferred to the ICU for advanced monitoring of vital functions. Early extubation and cessation of sedation were prioritized to facilitate immediate neurological assessment.

**Figure 1:**
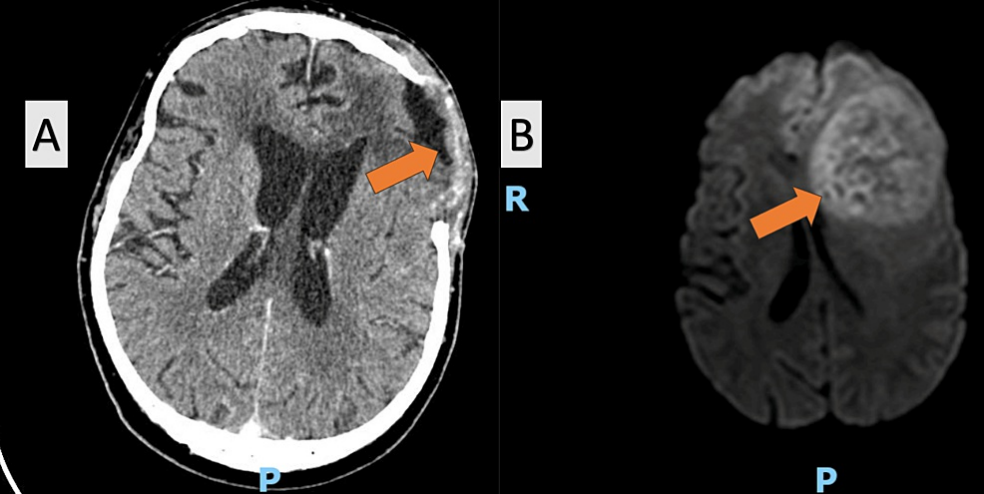
(A) Postoperative CT scan, (B) preoperative MRI scan

**Figure 2:**
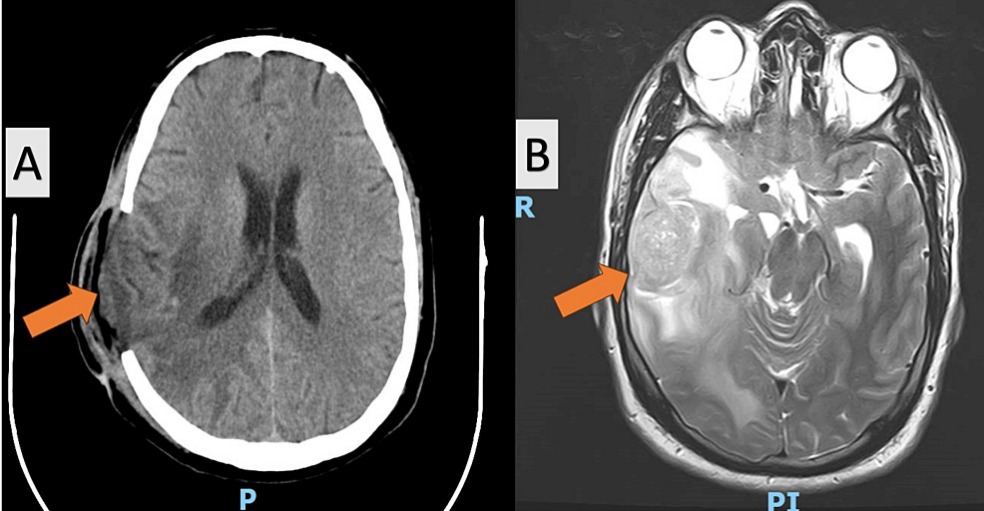
(A) Postoperative CT scan that shows no active hemorrhage, (B) preoperative MRI scan

**Figure 3:**
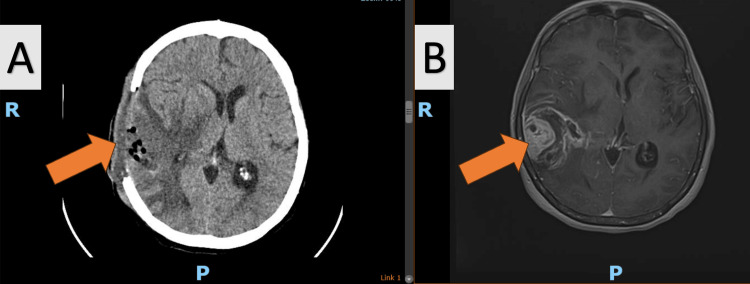
(A) Post-operative CT scan that shows tumoral resection and no active hemorrhage, (B) preoperative MRI scan that outlines the tumoral lesion

Postoperative care is critical for recovery, and here it included the initiation of neuromotor rehabilitation on the first postoperative day to promote recovery. Patients typically remained hospitalized for seven to 10 days postoperatively, until the surgical wound healed. A CT scan was conducted 48 hours postoperatively or if the patient's condition deteriorated (Figures 1, 2, 3). Patients were then referred to an oncologist for further evaluation and initiation of treatment based on histopathological findings. Specimens were sent to private laboratories to detect potential genetic abnormalities.

## Results

**Figure 4:**
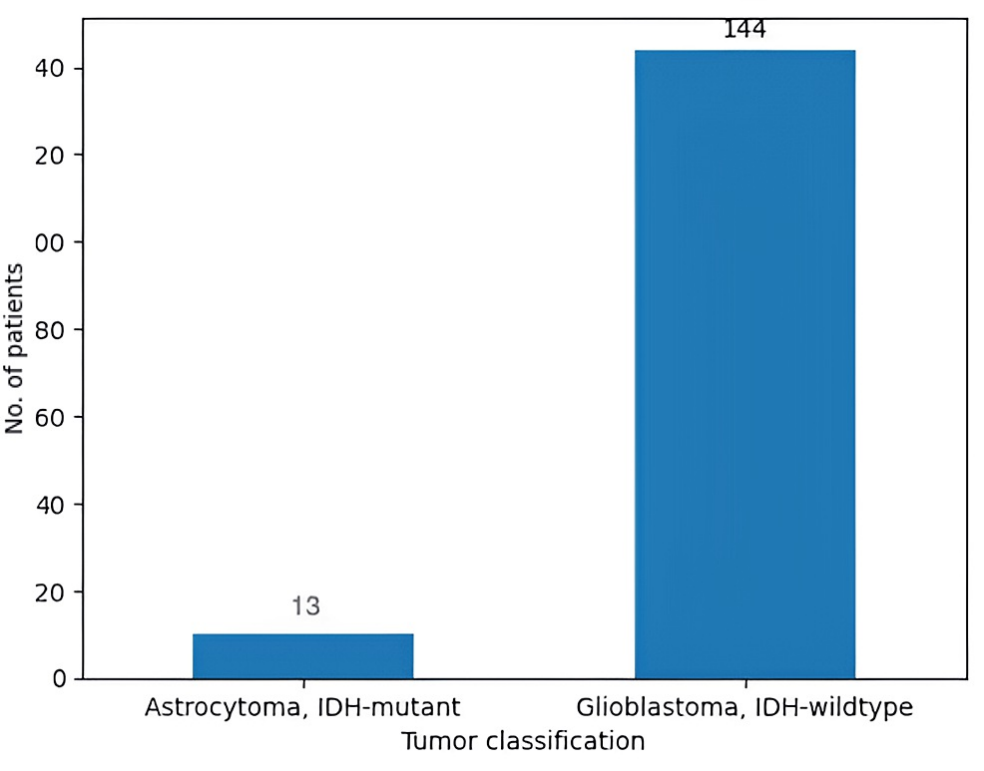
The initial number of patients in our study Out of the total of 157 patients with GTR, 144 had glioblastoma-wildtype, and the other 13 had astrocytomas. GTR: gross total resection

**Figure 5:**
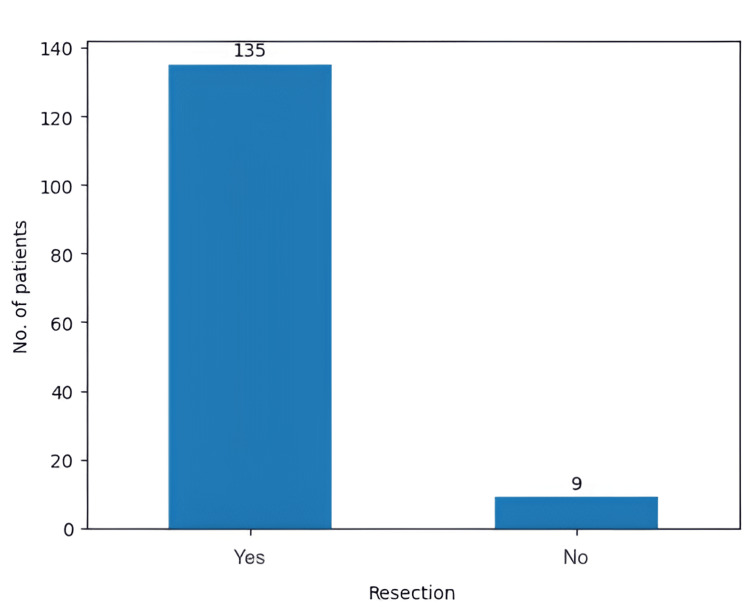
The total number of GTRs performed in our study GTR: gross total resection

Initially, our study was focused on the evolution of 157 patients with GBM who were treated over a time span of 12 years. However, the biopsies indicated that only 144 (91.7%) patients suffered from GBM, IDH-wildtype, and the other 13 (8.2%) had astrocytomas (Figure 4). We focused on the general aspects of GBM management, as well as recurrences, PFS, and OS. Out of the 144 patients, nine underwent only surgical biopsy, while the other 135 received a GTR (Figure 5).

**Figure 6:**
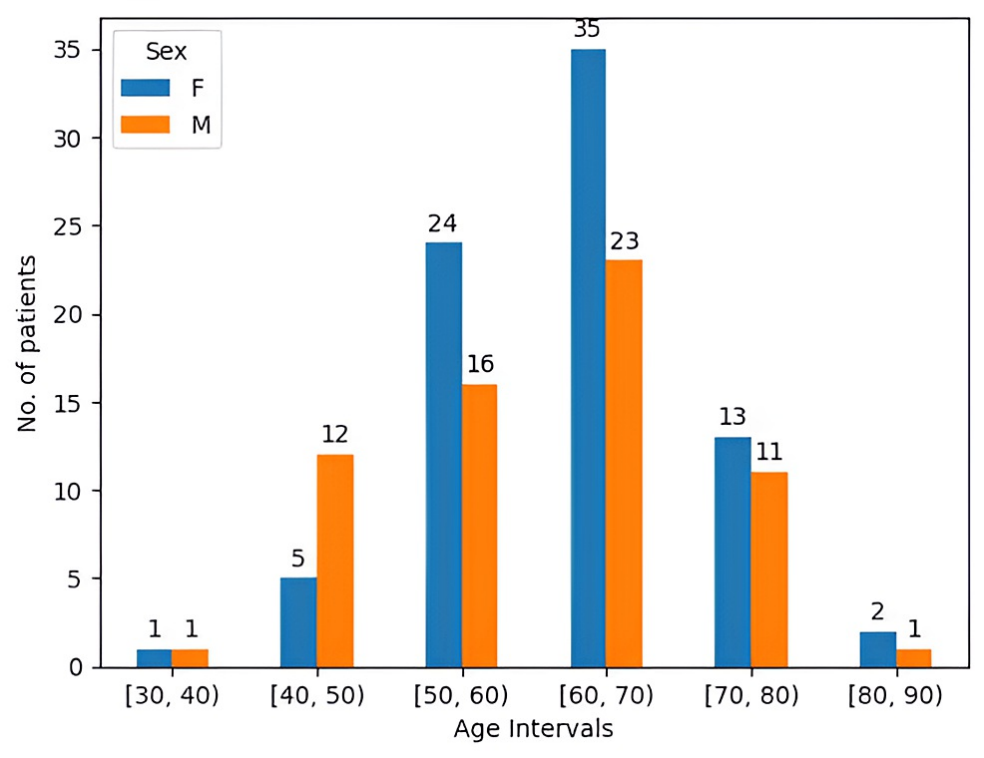
Sex distribution along six age intervals of 10 years

In our study, 64 of the 144 patients were male and 80 were female, leading to a male-to-female ratio of 0.8.:1 (Figure 6). Moreover, the age interval presented in our study was between 30-82 years, with the mean value being 60.7 years and the median 61 years.

**Figure 7:**
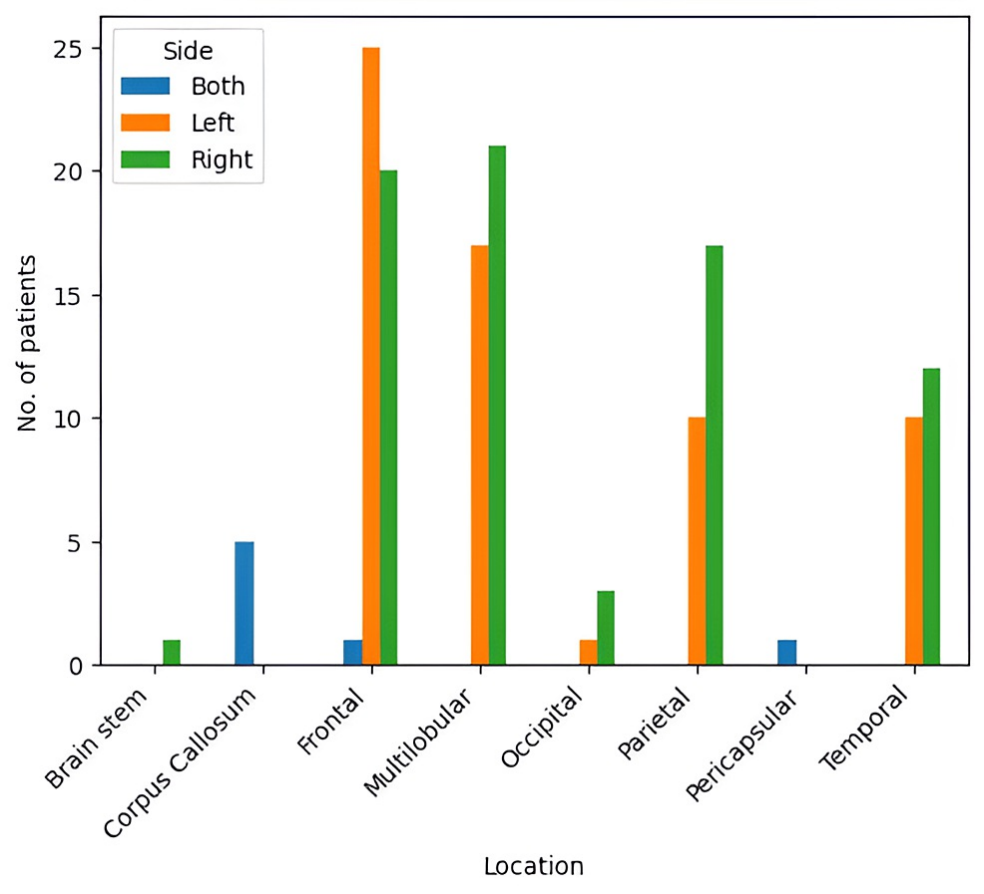
Localization prevalence of glioblastoma in the study group

**Figure 8:**
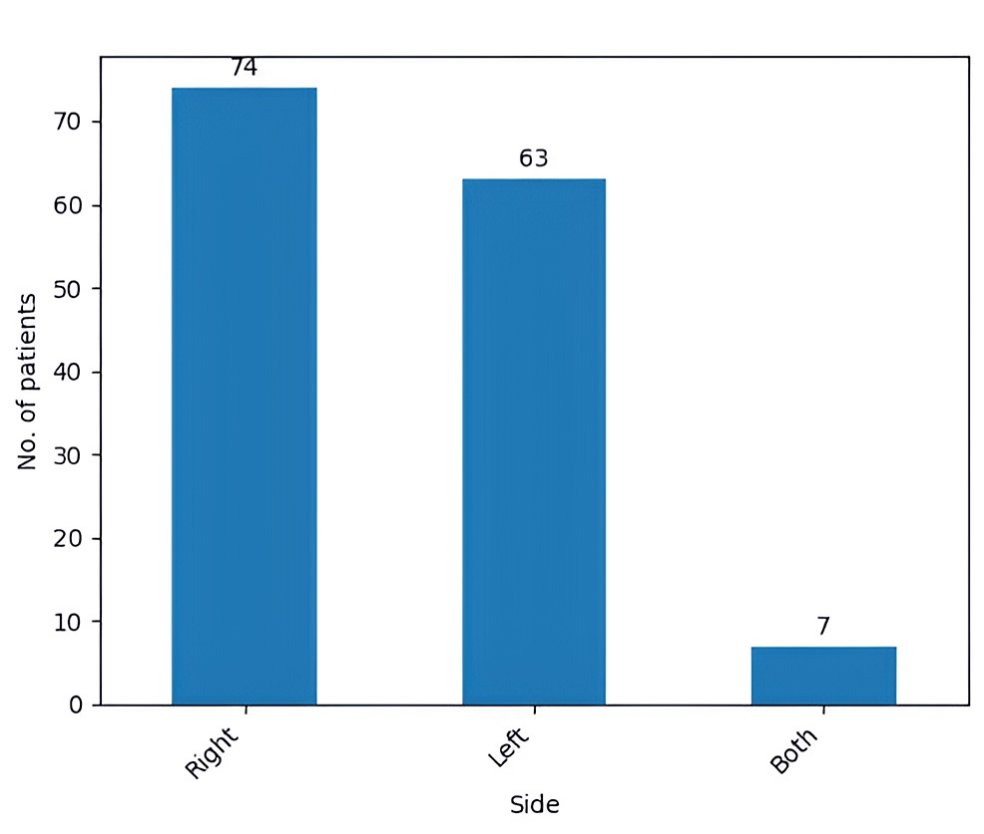
The side of the brain affected by the glioblastoma

Regarding the tumor characteristics, we evaluated which side of the brain was affected, and also the location of the tumor. Moreover, we evaluated the genetical characteristics of GBM, including MGMT promoter methylation status and EGFR amplifications. The most prevalent location was the frontal lobe, with 46 cases (31.9%), shortly followed by multilobular GBM, with 38 cases (26.3%), both accounting together for 84 (58.3%) out of the total 144 cases (Figure 7). Moreover, the right side was the most prevalent location for the tumoral process, accounting for 74 (51.3%) out of the 144 cases, with the left side being affected in 63 cases (43.7%). Both sides of the brain were affected in only seven (4.8%) out of the 144 patients (Figure 8).

**Figure 9:**
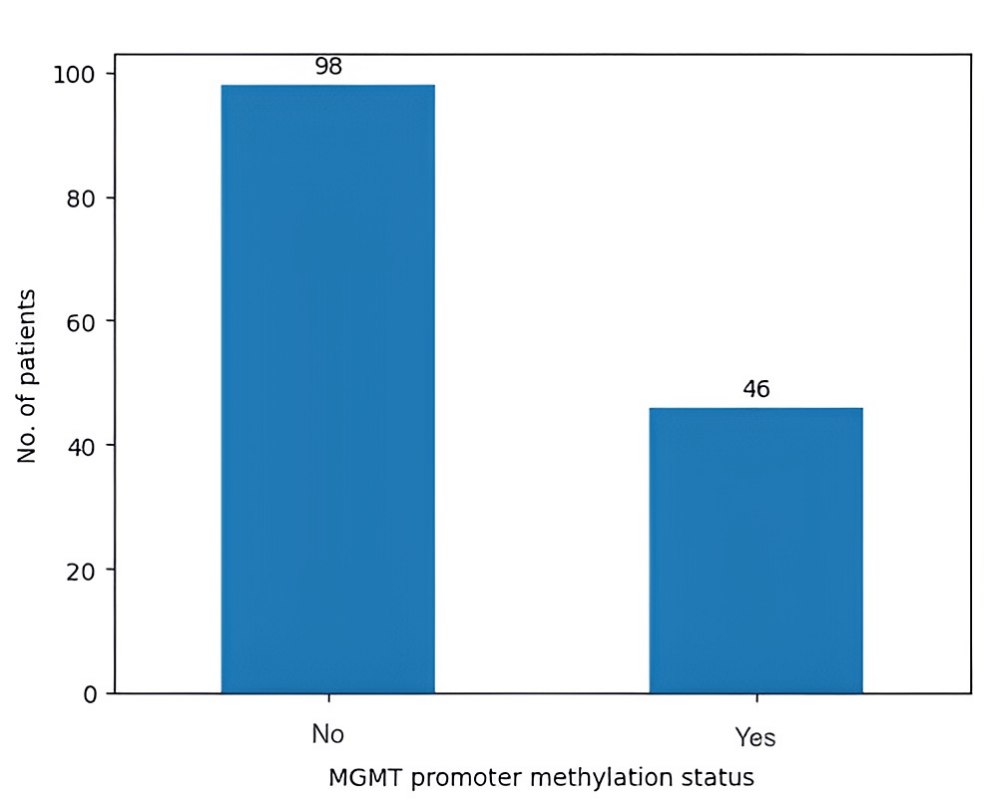
MGMT promoter methylation status MGMT: O6-methylguanine DNA methyltransferase

**Figure 10:**
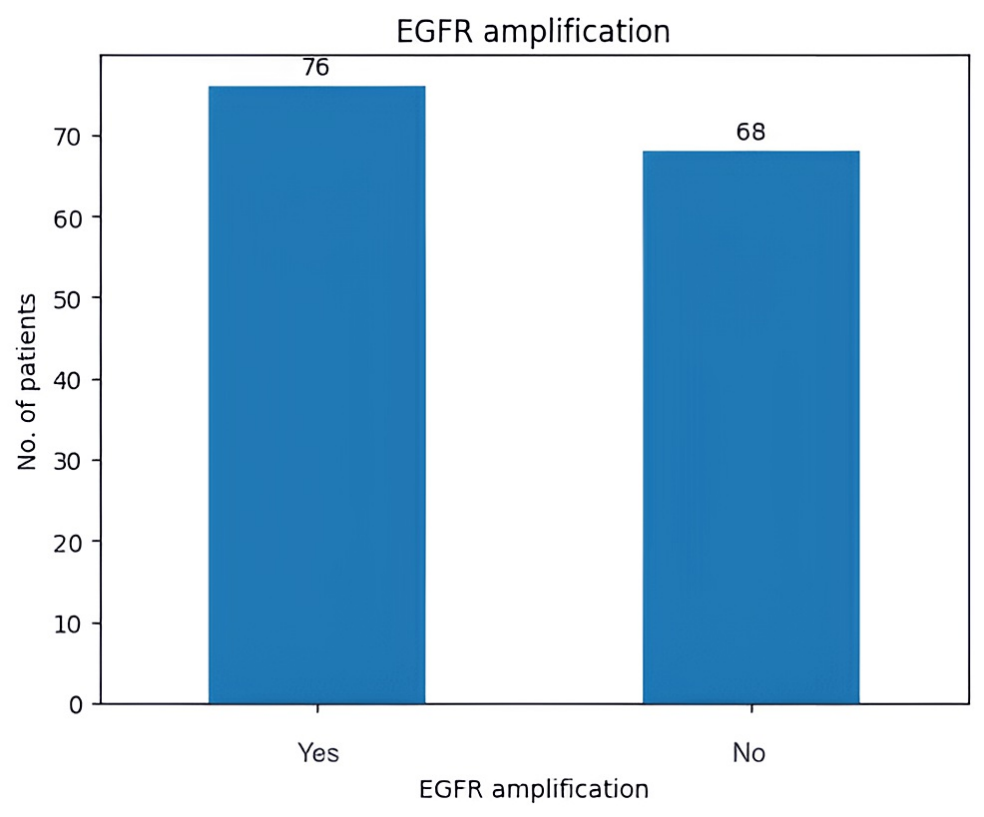
Diagram representing the number of patients with EGFR amplification EGFR: epidermal growth factor receptor

Another genetic aspect that was tested in our study group was MGMT promoter methylation status (Figure 9). In our study group, the MGMT promoter was methylated in 46 patients (31.9%). Moreover, EGFR was amplified in 76 (52.7%) patients (Figure 10).

**Figure 11:**
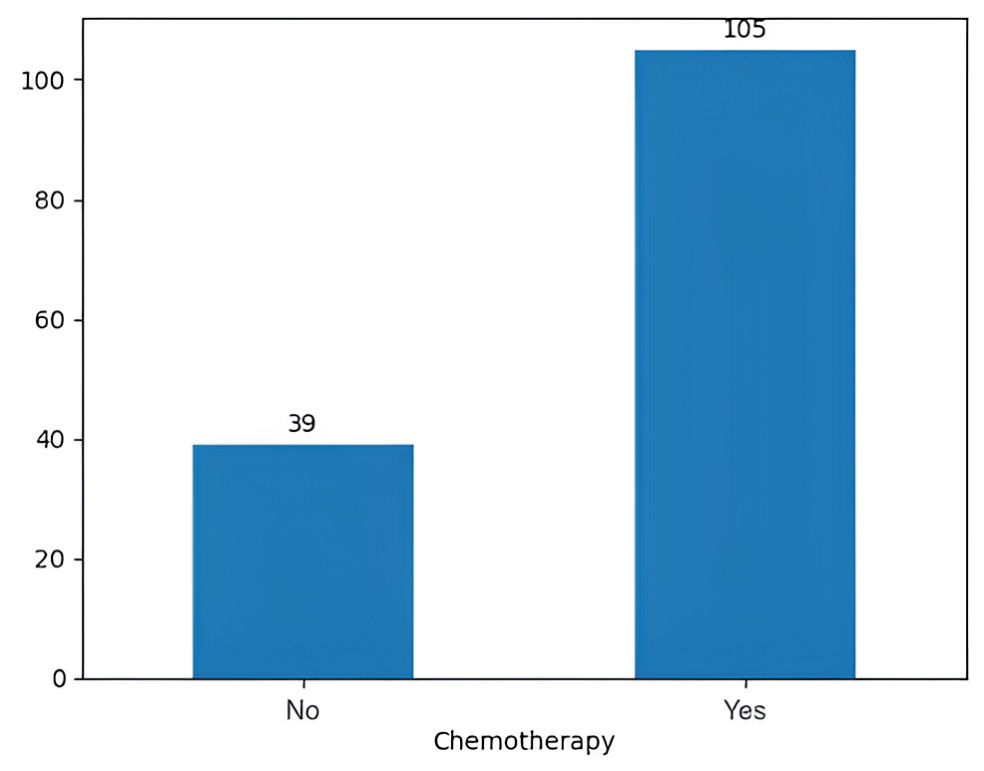
The number of patients who underwent chemotherapy

**Figure 12:**
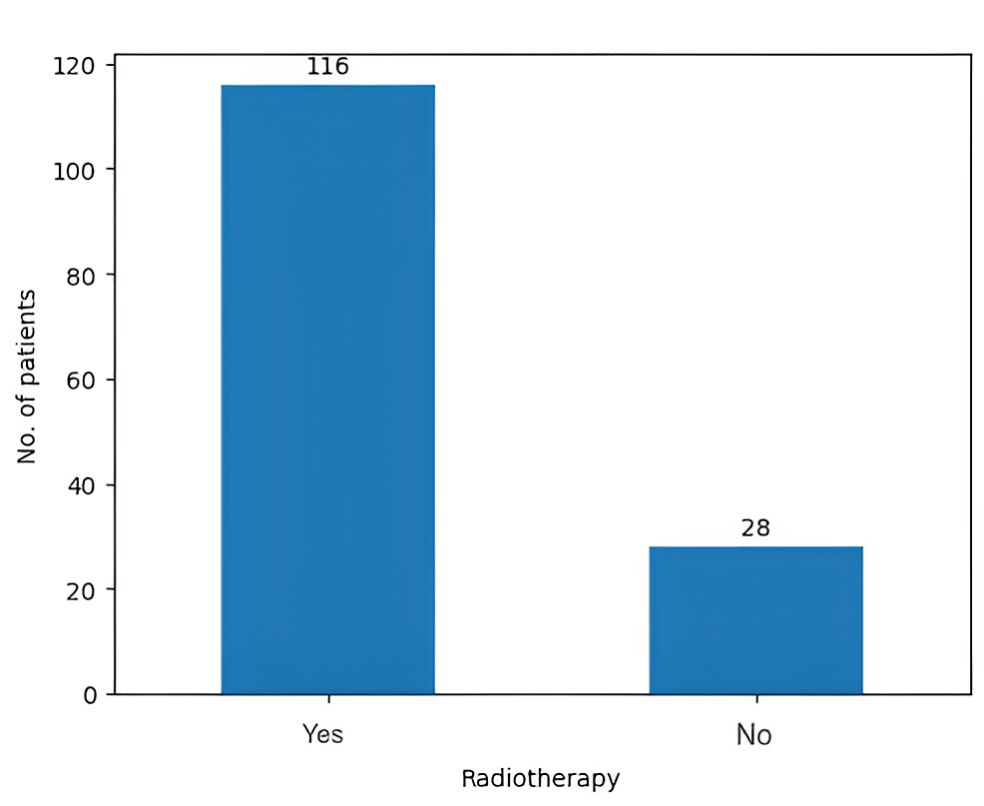
The number of patients who underwent radiotherapy

In our retrospective study, 105 (72.9%) patients opted for chemotherapy with TMZ (150 to 200 mg/m^2^, for five days every 28 days, six cycles), while the other 39 (27.1%) did not choose to undergo any chemotherapy (Figure 11). Moreover, 116 patients (80.5% ) underwent radiotherapy with a standard dose of 60 Gy, while the other 28 (19.4%) did not opt for radiotherapy (Figure 12). 

**Figure 13:**
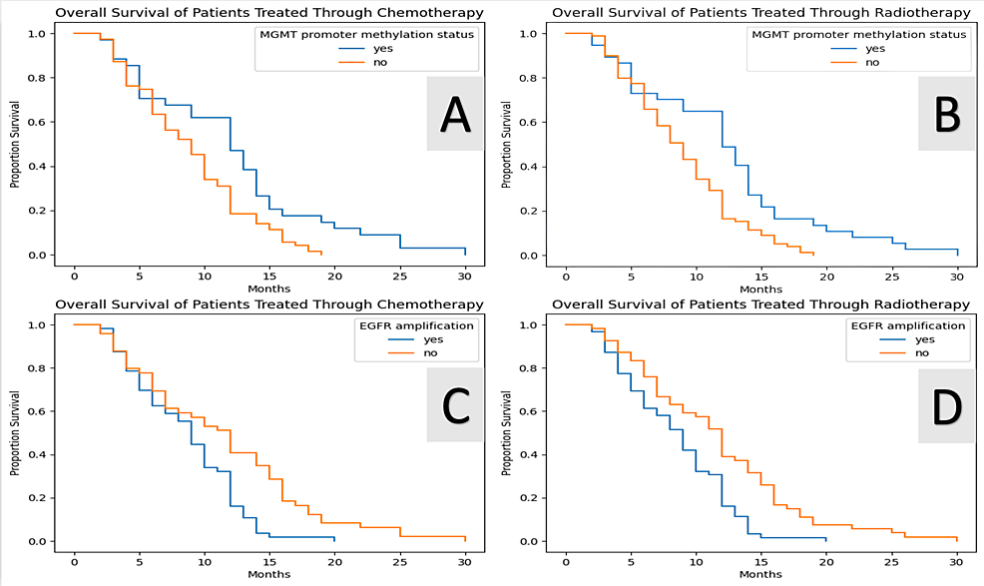
The effects of MGMT methylation status and EGFR amplification on the survival outcomes in patients who underwent chemotherapy and radiotherapy as seen in Kaplan-Meier survival outcome curves Using log-rank tests and Kaplan-Meier survival curves, we observed that overall survival (OS) significantly increased in patients with active methylation of the MGMT promoter, both in those who underwent chemotherapy (p<0.005) (A) and radiotherapy (p<0.005) (B). Similarly, a strong correlation was noted for the OS of patients without EGFR amplification who underwent chemotherapy (p<0.005) (C) and radiotherapy (p<0.005) (D). MGMT: O6-methylguanine DNA methyltransferase; EGFR: epidermal growth factor receptor

**Figure 14:**
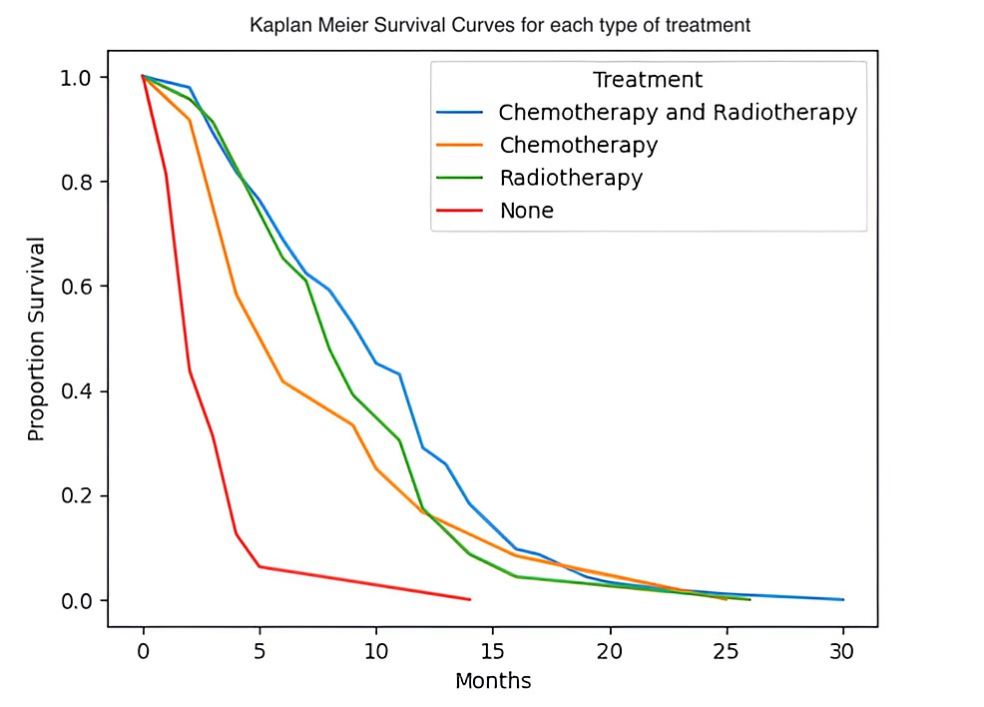
Kaplan-Meier survival curves for each type of treatment

Using log-rank tests and Kaplan-Meier survival curves, we were able to observe that OS increased in patients who had active methylation of the MGMT promoter in both patients who underwent chemotherapy and radiotherapy (p<0.005; Figure 13 A and Figure 13 B, respectively). Moreover, conducting the same tests, we observed the same significant connection of the OS in patients who had no amplification of the EGFR and underwent chemotherapy or radiotherapy (p<0.005; Figure 13 C and Figure 13 D, respectively). We also observed that patients who underwent both chemotherapy and radiotherapy (p<0.005) performed better OS-wise in comparison with patients who didn't undergo any type of adjuvant treatment (Figure 14). This was also the case for patients who underwent only chemotherapy or only radiotherapy, p=0.01 and p<0.005, respectively.

Pre-operative Karnofsky Performance Status (KPS) scale was employed in order to assess the overall survival of the patients. Once again, KPS proved to be of great relevance in the treatment of patients with GBM (Table 1). In our study, KPS 90 was the most prevalent, with 25 cases (17.3%), followed by an equal distribution of the other gradations. The mean KPS, in our case, was 56.59, with a median of 60. The distribution of our patients into three performance status groups was as follows: 34% represented High-Performance Status patients, 28.4% belonged to the Moderate Performance Status group, and 37.5% of the patients were part of the Low-Performance Status group. 

**Table 1:** Karnofsky Performance Status of the patients in our study High-Performance Status (80-100): 49 patients, Moderate-Performance Status (50-70): 41 patients, and Low-Performance Status (10-40): 54 patients

Karnofsky Performance Status	10	20	30	40	50	60	70	80	90	100
No. of patients	13	15	15	11	14	13	14	14	25	10

**Figure 15:**
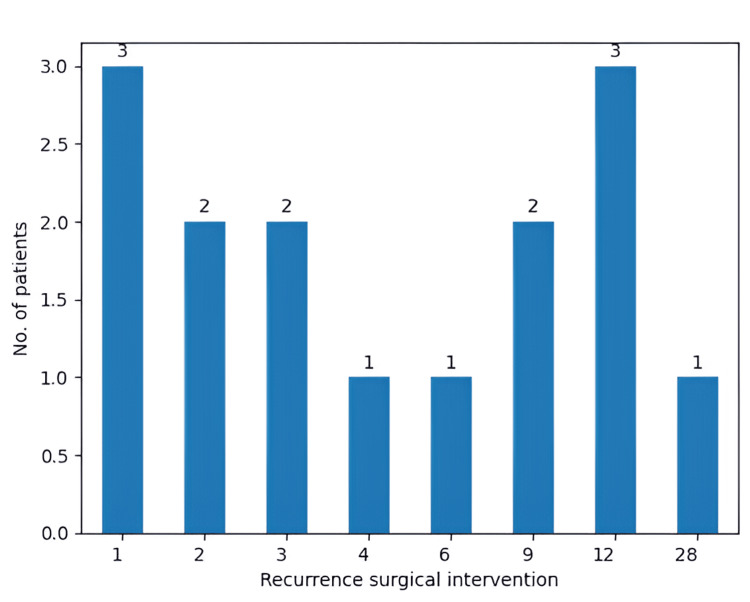
Number of months after which the patients underwent a second surgical intervention The X-axis stands for the time that has passed after the first surgical procedure, with time zero representing the time when the first surgical procedure happened

Moreover, we examined the number of recurrences that were treated surgically. In our study, only 15 patients (10.4%) underwent another surgical intervention. In the case of these patients, PFS had a mean of 5.87 months. Moreover, the mean OS of these patients was 10.8 months, with a minimum of two months and a maximum of 30 months (Figure 15).

**Figure 16:**
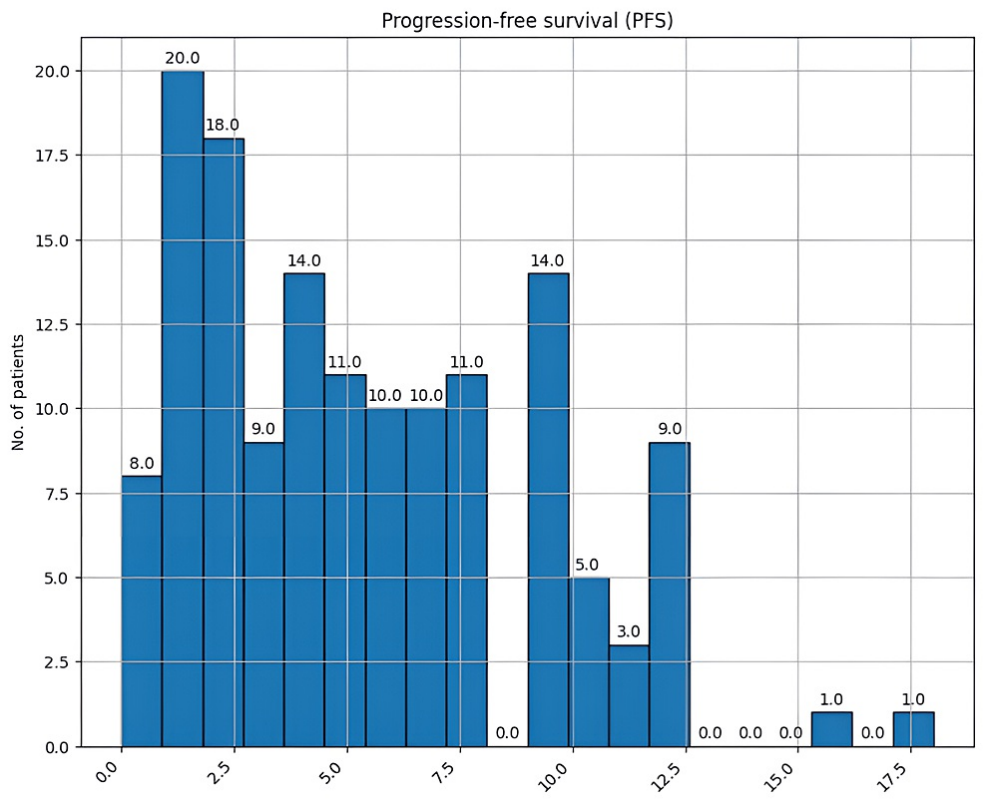
Progression-free survival (months) in our study

**Figure 17:**
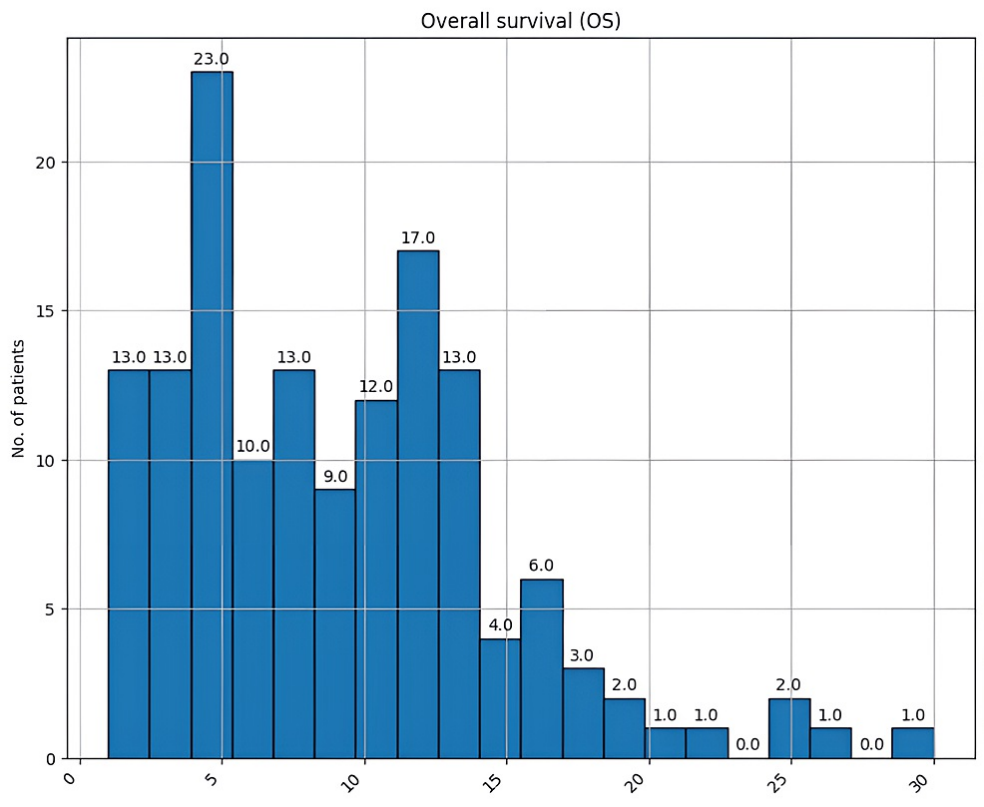
Overall survival (months) of the patients in this study

Lastly, crucial elements in GBM are PFS and OS, which in the case of this cancer are very low. PFS varied between zero and 18 months, with a mean of 5.3 months and a median of 5 months (Figure 16). OS varied between one and 30 months, with a mean of 9 months of survival and a median of 8.5 (Figure 17). 

## Discussion

We would like to start this Discussion section by enunciating the principal differences between this study’s results and the scientific literature. Our study differs from other similar studies through a different male-to-female ratio and lower average PFS and OS. 

Age represents one of the most relevant metrics in GBM patients because of its direct correlation with cellular aging, which usually leads to the development of GBM [13]. The median age in our study group of 144 patients was 61 years of age, which is slightly younger than the median scientific literature of 62 years of age for male and female patients, but does not represent a comparable difference due to the relatively small cohort [14,15].

The male-to-female ratio in our study was 0.8:1, lower than the usual ratio reported in the scientific literature, which shows a male tendency to develop the tumor more than females do as they age [16].

Pre-operative KPS score continues to be a crucial assessment tool, demonstrating significant relevance in patients with GBM [11,17]. 

A particularity of our country, that explains the lower-than-average median and mean OS, is that the patients often do not receive adjuvant treatment immediately after the surgical procedure. This particularity occurs because of multiple factors, such as the fact that there is no direct link between the neurosurgical service and the oncological service; they are separated from one another. Therefore, the oncological centers are full of patients looking for a cure, causing the oncologists to have a very low disponibility, creating long queues in the national oncological centers. Even though it might initially seem that this particularity could affect the results of this retrospective study misleadingly, the opposite is true. Adjuvant treatment is usually started four to five months after the surgical resection, meaning that by this point it is only used to treat the already recurred GBM, and not to prevent recurrences, thus becoming salvage therapy. Our study emphasizes the importance of the immediate institution of chemotherapy and radiotherapy right after resection, as proved in the standard Stupp protocol, which usually extends the OS of the patients [18].

MGMT promoter methylation status has become a key factor in determining prognosis and predicting response to TMZ [19,20]. Amplification of the EGFR gene is found in 57.4% of primary GBM patients and is linked to high levels of EGFR protein [21]. The literature supports that overexpressed wild-type EGFR and EGFR variant III (EGFRvIII) are not independent predictors of median OS in patients who did not undergo extensive tumor resection [22]. On the other hand, it was supported that MGMT promoter methylation is correlated with a prolonged PFS [23]. Our results show a direct correlation between a methylated MGMT promoter and an increase in OS in patients who underwent chemotherapy and radiotherapy, as well as an increase in OS for patients who underwent adjuvant therapy and had no EGFR amplification, findings which support the current literature.

Circulating extracellular vesicles (EVs) represent an exceptionally promising biomarker class detectable in the blood of patients diagnosed with GBM. These membrane-bound nanoparticles, released by virtually all cell types, contain DNA, RNA, proteins, and lipids that reflect the identity and molecular state of their source cells. They represent a novel, minimally invasive diagnostic method [24,25]. Besides novel diagnostic methods for GBM, the international scientific community is also seeking novel therapeutic agents. 

Lastly, it is important to observe that the mean PFS and OS of the group that had a second surgical intervention is slightly higher than the general mean PFS and OS. Therefore, it is safe to say that it should be of great importance to consider a second surgical intervention when it is possible, since a problem that arises frequently with GBM is the fact that the tumor proliferates in the healthy tissue, and it is almost impossible to perform a complete resection of the tumor. Therefore, our study confirms that surgery as a salvage therapy is a reliable treatment strategy. Also, a meta-analysis performed by Lu et al. further reinforces the point that a second surgical procedure points toward a greater OS [26]. A multi-center retrospective study with 1032 patients concluded that surgery for recurrent GBM may be beneficial in some instances with acceptable morbidity rates and satisfactory local control and survival benefits [27]. Stereotactic radiotherapy (SRT), on the other hand, was identified as another viable salvage treatment option offering satisfactory local control and survival advantages [28]. Furthermore, apatinib plus TMZ was seen to provide effective salvage therapy with manageable toxicities; moreover, it did not reduce bevacizumab's effectiveness either [29]. However, salvage therapy still remains a debated topic among the medical community worldwide [1,30,31].

The weaknesses of our retrospective study are as follows. Firstly, a small number of patients were selected. Secondly, because genetic information is not assessed in our institution, there is a slight possibility that some results might be false positives or negatives. Another weakness is the lack of a direct link between the neurosurgical department and the oncological department in our country. The severe acute respiratory syndrome coronavirus 2 (SARS‑CoV‑2) pandemic has also delayed oncological and surgical treatment in the case of some of the patients. Other disadvantages include the fact that not all patients who underwent surgery for GBM in our institution during that period were included in this study because not all of them were cooperative and follow-up was difficult to obtain. Lastly, the post-operative KPS score was not assessed because patients did not receive a pre-operative MRI tractography; therefore, assessing this score post-surgery would not be highly accurate.

## Conclusions

Our retrospective study of 144 GBM patients treated over 12 years provides significant insights into the factors affecting prognosis and survival outcomes. Our findings reaffirm the importance of the KPS as a reliable predictor of patient outcomes in GBM. The study also underscores the crucial role of immediate postoperative adjuvant therapy in extending PFS and OS. Genetic factors such as MGMT promoter methylation and EGFR gene amplification were highlighted as key determinants of prognosis and treatment response. Despite the challenges posed by the complex and infiltrative nature of GBM, our research suggests that timely and comprehensive treatment, including potential second surgical interventions, can improve patient outcomes. This study emphasizes the need for coordinated care and timely access to adjuvant therapies to enhance survival rates in GBM patients.
